# The type IV polymorph of KEu(PO_3_)_4_

**DOI:** 10.1107/S1600536809048788

**Published:** 2009-11-21

**Authors:** Abdelghani Oudahmane, Mohamed Daoud, Boumediene Tanouti, Daniel Avignant, Daniel Zambon

**Affiliations:** a Université Cadi Ayyad, Laboratoire de Physico-Chimie des Matériaux et Environnement, Faculté des Sciences Semlalia, Département de Chimie, BP 2390, 40000, Marrakech, Morocco; b Université Blaise Pascal, Laboratoire des Matériaux Inorganiques, UMR CNRS 6002, 24 Avenue des Landais, 63177 Aubière, France

## Abstract

Single crystals of KEu(PO_3_)_4_, potassium europium(III) polyphosphate, were obtained by solid-state reactions. This monoclinic form is the second polymorph described for this composition and belongs to type IV of long-chain polyphosphates with general formula *A* ^I^ *B* ^III^(PO_3_)_4_. It is isotypic with its KEr(PO_3_)_4_ and KDy(PO_3_)_4_ homologues. The crystal structure is built of infinite helical chains of corner-sharing PO_4_ tetra­hedra with a repeating unit of eight tetra­hedra. These chains are further linked by isolated EuO_8_ square anti­prisms, forming a three-dimensional framework. The K^+^ ions are located in pseudo-hexa­gonal channels running along [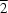
01] and are surrounded by nine O atoms in a distorted environment.

## Related literature

Besides crystals of the title compound, crystals of the type III polymorph (Hu *et al.*, 1984 ▶)) have also been obtained. For isotypic *AB*(PO_3_)_4_ structures, where *A* is an alkali metal, Tl or NH_4_^+^, and *B* is a rare earth element, see: Palkina *et al.* (1977 ▶) for TlNd; Maksimova *et al.* (1978 ▶) for RbNd; Dago *et al.* (1980 ▶) for KEr; Maksimova *et al.* (1981 ▶) for CsNd; Maksimova *et al.* (1982 ▶) for RbHo; Horchani *et al.* (2004 ▶) for RbEr; Rekik *et al.* (2004 ▶) for KGd; Naïli & Mhiri (2005 ▶) for CsGd; Ben Zarkouna *et al.* (2006 ▶) for (NH_4_)Gd; Khlissa & Férid (2006 ▶) for RbTb; Ettis *et al.* (2006 ▶) for RbGd; Chehimi-Moumen & Férid (2007 ▶) for KDy; Horchani-Naifer & Férid (2007 ▶) for CsPr; Zhu *et al.* (2009 ▶) for CsEu. For a review on the crystal chemisty of polyphosphates, see: Durif (1995 ▶). Jaouadi *et al.* (2003 ▶) have discussed the main crystal chemical characteristics of the seven *AB*(PO_3_)_4_ structure types. For applications of rare earth polyphosphates, see: Rashchi & Finch (2000 ▶); Barsukov *et al.* (2004 ▶). For general background, see: Porai-Koshits & Aslanov (1972 ▶). For ionic radii, see: Shannon (1976 ▶).

## Experimental

### 

#### Crystal data

KEu(PO_3_)_4_
*M* *_r_* = 506.94
Monoclinic, 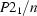

*a* = 10.3723 (1) Å
*b* = 8.9721 (1) Å
*c* = 10.8320 (1) Å
β = 106.053 (1)°
*V* = 968.73 (2) Å^3^
*Z* = 4
Mo *K*α radiation
μ = 7.63 mm^−1^
*T* = 296 K
0.12 × 0.11 × 0.10 mm

#### Data collection

Bruker APEXII CCD diffractometer
Absorption correction: multi-scan (*SADABS*; Bruker, 2008 ▶) *T*_min_ = 0.466, *T*_max_ = 0.513
24299 measured reflections
6201 independent reflections
5023 reflections with *I* > 2σ(*I*)
*R*_int_ = 0.045

#### Refinement

*R*[*F* ^2^ > 2σ(*F* ^2^)] = 0.030
*wR*(*F* ^2^) = 0.072
*S* = 1.04
6201 reflections
163 parameters
Δρ_max_ = 1.72 e Å^−3^
Δρ_min_ = −2.04 e Å^−3^

### 

Data collection: *APEX2* (Bruker, 2008 ▶); cell refinement: *SAINT* (Bruker, 2008 ▶); data reduction: *SAINT*; program(s) used to solve structure: *SHELXS97* (Sheldrick, 2008 ▶); program(s) used to refine structure: *SHELXL97* (Sheldrick, 2008 ▶); molecular graphics: *DIAMOND* (Brandenburg, 1999 ▶); software used to prepare material for publication: *SHELXTL* (Sheldrick, 2008 ▶).

## Supplementary Material

Crystal structure: contains datablocks I, global. DOI: 10.1107/S1600536809048788/wm2282sup1.cif

Structure factors: contains datablocks I. DOI: 10.1107/S1600536809048788/wm2282Isup2.hkl

Additional supplementary materials:  crystallographic information; 3D view; checkCIF report

## Figures and Tables

**Table 1:** Selected bond lengths (Å)

P1—O7	1.480 (2)
P1—O4^i^	1.483 (2)
P1—O12	1.588 (2)
P1—O3^ii^	1.5968 (19)
P2—O10^iii^	1.4795 (19)
P2—O5	1.483 (2)
P2—O11^iii^	1.6015 (18)
P2—O3^iv^	1.602 (2)
P3—O6	1.482 (2)
P3—O2	1.4873 (19)
P3—O11	1.6005 (19)
P3—O1	1.6033 (19)
P4—O9	1.4784 (19)
P4—O8^v^	1.484 (2)
P4—O1	1.601 (2)
P4—O12^iii^	1.601 (2)

Symmetry codes: (i) 
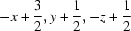
; (ii) 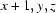; (iii) 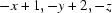; (iv) 
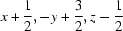
; (v) 
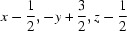
.

**Fig. 1.**
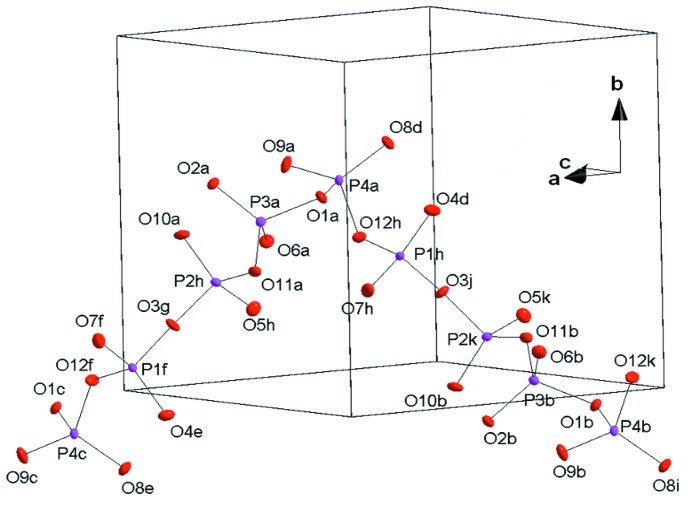
ORTEP-3 view of the repeating unit with eight PO4 tetrahedra, leading to helical (PO3)∞ chains. Displacement ellipsoids are drawn at the 50 % probability level. Symmetry codes: (a) -1/2+x, 3/2-y, 1/2+z; (b) -1+x, -1+y, z; (c) x, -1+y, 1+z; (d) -1+x, y, z; (e) -1/2+x, 1/2-y, 1/2+z; (f) 3-x, 1-y, 1-z; (g) 2-x, 1-y, 1-z; (h) 5/2-x, -1/2+y, 1/2-z; (i) -3/2+x, 1/2-y, -1/2+z; (j) 3/2-x, -1/2+y, 1/2-z; (k) 2-x, 1-y, -z.

**Fig. 2.**
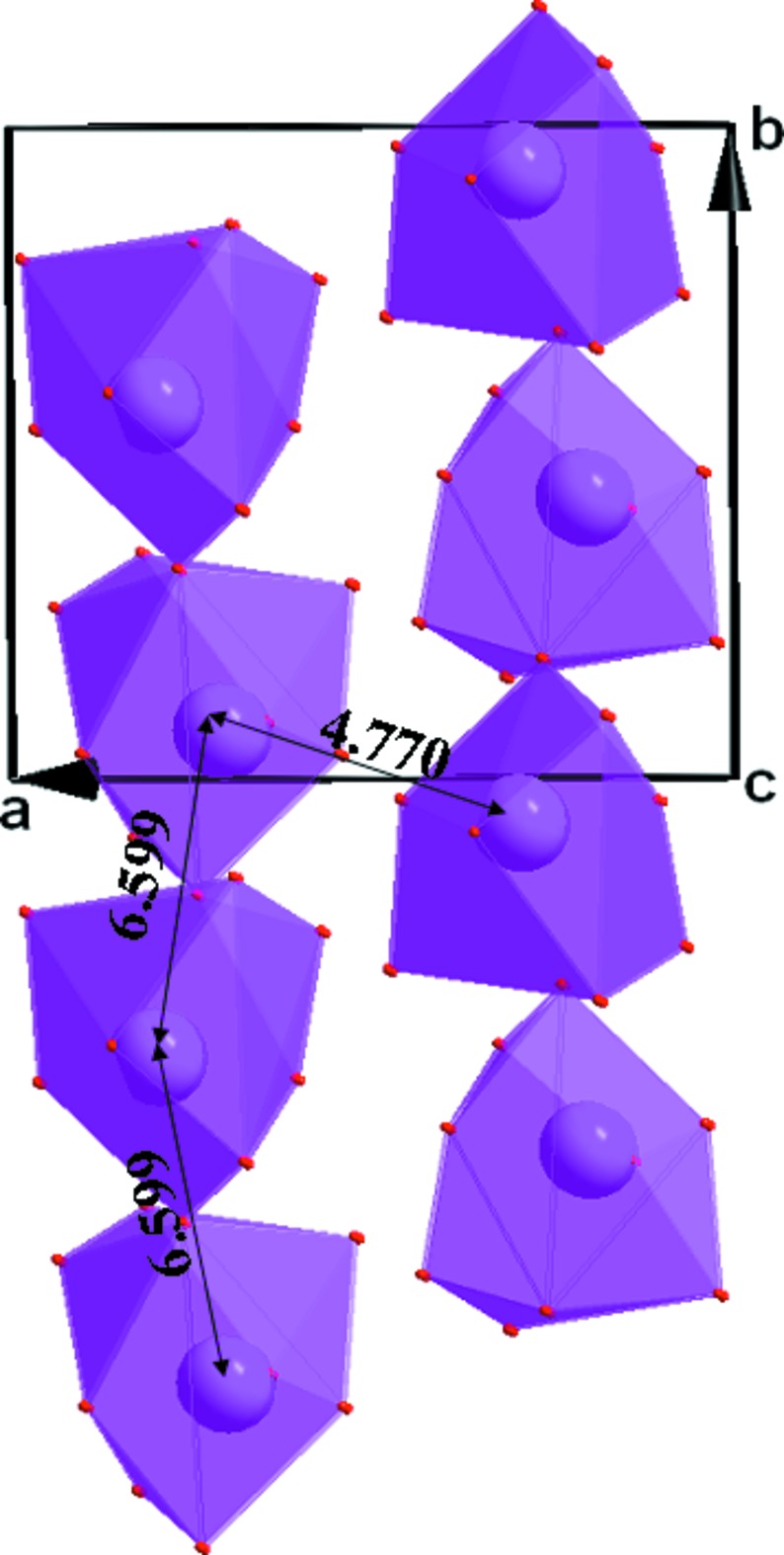
Partial view of infinite chains of corner-sharing KO9 polyhedra.

## supplementary crystallographic information

### Comment

Rare earth polyphosphates are interesting materials and bear potential applications (Rashchi & Finch, 2000; Barsukov *et al.*, 2004). The title compound is a member of a large family of polyphosphates with general formula *A*^I^*B*^III^(PO_3_)_4_ (where *A*^I^ is a monovalent cation: Li, Na, K, Rb, Cs, Tl, NH_4_, Ag and *B*^III^ is a trivalent cation: Ln,Y, Bi). It is now well known that these compounds are classified into seven structural types usually labelled by roman numerals from I to VII. A short recapitulation of the main crystal chemical characteristics of these seven structural types has recently been given by Jaouadi *et al.* (2003). The KEu(PO_3_)_4_ polymorph described in this article belongs to the IV structural type.

In the crystal structure the Eu^3+^ ion is eight-coordinated by the oxygen atoms and its 8-coordination polyhedron is better described as a square antiprism than a dodecahedron according to the criteria of Porai-Koshits & Aslanov (1972) (δ_1_ = 10.37°, δ_2_ = 10.85°, δ_3_ = 47.97°, δ_4_ = 53.54°). The Eu—O distances range from 2.3199 (19) Å to 2.4827 (19) Å with an average <Eu—O> distance of 2.399 Å that is slightly shorter than the sum of the ionic radii *i.e.* 2.466 Å (Shannon, 1976). The structure of this type IV polymorph is built of infinite helical chains of corner-sharing PO_4_ tetrahedra further linked by isolated EuO_8_ square antiprisms. The (PO_3_)_∞_ chains exhibit a repeating unit of eight PO_4_ tetrahedra (Fig. 1) and are running along the [101] direction. The three-dimensional framework resulting from the edge-sharing between the PO_4_ tetrahedra and the EuO_8_ square antiprisms exhibits pseudo hexagonal channels where the K^+^ ions reside. The K^+^ ion is 9-coordinated by oxygen atoms with distances ranging from 2.789 (2) Å to 3.370 (3) Å. By sharing corners, the KO_9_ coordination polyhedra form corrugated chains running along the [010] direction (Fig. 2). Whereas the K atoms are separated by 6.599 (2) Å within the chain, the shortest K—K distance in the structure, 4.770 (2) Å, occurs between two adjacent (KO_9_)_∞_ chains. This shortest distance corresponds to the separation between two K^+^ ions within the channels of the structure running along the [201] direction. This separation distance *A*^I^—*A*^I^ is strongly dependent on the nature of the *A*^I^ element and decreases as the size of the *A*^I^ element increases. For instance, in the *A*^I^Gd(PO_3_)_4_ homologue series, where *A*^I^ = K, Rb, Cs, this *A*^I^—*A*^I^ shortest distance varies from 4.801 Å for K to 4.211 Å for Cs (4.524 Å for Rb). For CsEu(PO_3_)_4_ the shortest Cs—Cs distance is equal to 4.237Å (Zhu *et al.* 2009).

For isotypic *AB*(PO_3_)_4_ structures, where *A* is an alkali metal, Tl or NH_4_^+^, and *B* is a rare earth element, see: Palkina *et al.* (1977) for TlNd, Maksimova *et al.* (1978) for RbNd, Dago *et al.* (1980) for KEr, Maksimova *et al.* (1981) for CsNd, Maksimova *et al.* (1982) for RbHo, Horchani *et al.* (2004) for RbEr, Rekik *et al.* (2004) for KGd, Naïli & Mhiri (2005) for CsGd, Ben Zarkouna *et al.* (2006) for (NH_4_)Gd, Khlissa & Férid (2006) for RbTb, Ettis *et al.* (2006) for RbGd, Chehimi-Moumen & Férid (2007) for KDy, Horchani-Naifer & Férid (2007) for CsPr, and Zhu *et al.* (2009) for CsEu. For a review on the crystal chemisty of polyphosphates, see: Durif (1995).

### Experimental

Crystals of the title compound were synthesized by reacting Eu_2_O_3_ with (NH_4_)H_2_PO_4_ and K_2_CO_3_ in a platinum crucible. A mixture of these reagents in the molar ratio 34:57:9 was used for the synthesis. The mixture was heated at 473 K for 6 h, then at 573 K for 6 h and finally at 873 K for 24 h. The furnace was then cooled down first to 773 K at the rate of 2 K^.^h^-1^ and then to room temperature at the rate of K^.^h^-1^. Single crystals were extracted from the batch by washing with hot water. Besides crystals of the title compound, crystals of the type III polymorph (Hu *et al.*, 1984)) have also been obtained.

### Refinement

The highest residual peak in the final difference Fourier map was located 0.68 Å from atom Eu and the deepest hole was located 0.45 Å from atom K.

### Crystal data

**Table:**

KEu(PO_3_)_4_	*F*(000) = 952
*M**_r_* = 506.94	*D*_x_ = 3.476 Mg m^−^^3^
Monoclinic, *P*2_1_/*n*	Mo *K*α radiation, λ = 0.71073 Å
Hall symbol: -P 2yn	Cell parameters from 6203 reflections
*a* = 10.3723 (1) Å	θ = 2.6–40.6°
*b* = 8.9721 (1) Å	µ = 7.63 mm^−^^1^
*c* = 10.8320 (1) Å	*T* = 296 K
β = 106.053 (1)°	Hexagonal prism, colourless
*V* = 968.73 (2) Å^3^	0.12 × 0.11 × 0.10 mm
*Z* = 4	

### Data collection

**Table:**

Bruker APEXII CCD diffractometer	6201 independent reflections
Radiation source: fine-focus sealed tube	5023 reflections with *I* > 2σ(*I*)
graphite	*R*_int_ = 0.045
Detector resolution: 8.3333 pixels mm^-1^	θ_max_ = 40.6°, θ_min_ = 3.1°
ω and φ scans	*h* = −18→18
Absorption correction: multi-scan (*SADABS*; Bruker, 2008)	*k* = −16→16
*T*_min_ = 0.466, *T*_max_ = 0.513	*l* = −19→5
24299 measured reflections	

### Refinement

**Table:**

Refinement on *F*^2^	0 constraints
Least-squares matrix: full	Primary atom site location: structure-invariant direct methods
*R*[*F*^2^ > 2σ(*F*^2^)] = 0.030	Secondary atom site location: difference Fourier map
*wR*(*F*^2^) = 0.072	*w* = 1/[σ^2^(*F*_o_^2^) + (0.0311*P*)^2^ + 1.1689*P*] where *P* = (*F*_o_^2^ + 2*F*_c_^2^)/3
*S* = 1.04	(Δ/σ)_max_ = 0.002
6201 reflections	Δρ_max_ = 1.72 e Å^−^^3^
163 parameters	Δρ_min_ = −2.04 e Å^−^^3^
0 restraints	

### Special details

**Table:**

Geometry. All e.s.d.'s (except the e.s.d. in the dihedral angle between two l.s. planes) are estimated using the full covariance matrix. The cell e.s.d.'s are taken into account individually in the estimation of e.s.d.'s in distances, angles and torsion angles; correlations between e.s.d.'s in cell parameters are only used when they are defined by crystal symmetry. An approximate (isotropic) treatment of cell e.s.d.'s is used for estimating e.s.d.'s involving l.s. planes.
Refinement. Refinement of *F*^2^ against ALL reflections. The weighted *R*-factor *wR* and goodness of fit *S* are based on *F*^2^, conventional *R*-factors *R* are based on *F*, with *F* set to zero for negative *F*^2^. The threshold expression of *F*^2^ > σ(*F*^2^) is used only for calculating *R*-factors(gt) *etc*. and is not relevant to the choice of reflections for refinement. *R*-factors based on *F*^2^ are statistically about twice as large as those based on *F*, and *R*- factors based on ALL data will be even larger.

### Fractional atomic coordinates and isotropic or equivalent isotropic displacement parameters (Å^2^)

**Table:**

	*x*	*y*	*z*	*U*_iso_*/*U*_eq_	
K	0.79467 (11)	0.57090 (14)	0.04227 (10)	0.0395 (2)	
Eu	0.500385 (11)	0.772652 (13)	0.184899 (12)	0.00645 (3)	
P1	0.85428 (6)	0.90558 (7)	0.24010 (7)	0.00615 (10)	
P2	0.54001 (6)	0.82848 (7)	−0.14102 (7)	0.00607 (10)	
P3	0.24938 (6)	1.02436 (7)	0.22927 (6)	0.00609 (10)	
P4	0.17657 (6)	0.89469 (7)	−0.01962 (6)	0.00626 (10)	
O1	0.14215 (17)	0.9549 (2)	0.10673 (19)	0.0085 (3)	
O2	0.35389 (19)	0.9131 (2)	0.29039 (19)	0.0093 (3)	
O3	−0.02278 (19)	0.7943 (2)	0.2518 (2)	0.0103 (3)	
O4	0.6017 (2)	0.5368 (2)	0.1754 (2)	0.0117 (3)	
O5	0.5648 (2)	0.7615 (2)	−0.0114 (2)	0.0117 (3)	
O6	0.17180 (19)	1.0919 (2)	0.3112 (2)	0.0106 (3)	
O7	0.73783 (19)	0.8192 (2)	0.2547 (2)	0.0134 (4)	
O8	0.5691 (2)	0.7074 (2)	0.4090 (2)	0.0114 (3)	
O9	0.31707 (19)	0.8419 (2)	0.0175 (2)	0.0121 (3)	
O10	0.53947 (19)	1.0334 (2)	0.1765 (2)	0.0121 (3)	
O11	0.31589 (17)	1.1548 (2)	0.1668 (2)	0.0092 (3)	
O12	0.8309 (2)	0.9497 (2)	0.0936 (2)	0.0125 (3)	

### Atomic displacement parameters (Å^2^)

**Table:**

	*U*^11^	*U*^22^	*U*^33^	*U*^12^	*U*^13^	*U*^23^
K	0.0363 (5)	0.0581 (6)	0.0243 (4)	0.0047 (4)	0.0085 (4)	−0.0045 (4)
Eu	0.00596 (4)	0.00617 (4)	0.00701 (5)	0.00051 (3)	0.00144 (3)	0.00084 (4)
P1	0.0054 (2)	0.0053 (2)	0.0068 (2)	−0.00011 (17)	0.0002 (2)	0.00105 (19)
P2	0.0060 (2)	0.0053 (2)	0.0071 (2)	−0.00059 (17)	0.0021 (2)	−0.00025 (19)
P3	0.0057 (2)	0.0065 (2)	0.0062 (3)	0.00066 (17)	0.0020 (2)	−0.00024 (19)
P4	0.0052 (2)	0.0077 (2)	0.0050 (2)	−0.00047 (17)	0.00004 (19)	0.00081 (19)
O1	0.0071 (6)	0.0106 (7)	0.0078 (7)	−0.0029 (5)	0.0023 (6)	−0.0027 (6)
O2	0.0094 (7)	0.0093 (7)	0.0087 (8)	0.0032 (5)	0.0015 (6)	0.0013 (6)
O3	0.0108 (7)	0.0104 (7)	0.0101 (8)	0.0053 (6)	0.0036 (6)	0.0047 (6)
O4	0.0163 (8)	0.0086 (7)	0.0117 (8)	0.0013 (6)	0.0063 (7)	0.0015 (6)
O5	0.0135 (8)	0.0155 (8)	0.0074 (8)	0.0021 (6)	0.0048 (7)	0.0014 (6)
O6	0.0114 (7)	0.0126 (7)	0.0095 (8)	0.0022 (6)	0.0059 (7)	−0.0018 (6)
O7	0.0073 (7)	0.0148 (8)	0.0173 (10)	−0.0029 (6)	0.0019 (7)	0.0051 (7)
O8	0.0114 (7)	0.0125 (8)	0.0093 (8)	0.0046 (6)	0.0011 (6)	0.0038 (6)
O9	0.0083 (7)	0.0193 (9)	0.0080 (8)	0.0053 (6)	0.0011 (6)	0.0024 (7)
O10	0.0099 (7)	0.0060 (6)	0.0212 (10)	0.0017 (5)	0.0059 (7)	0.0020 (7)
O11	0.0060 (6)	0.0077 (6)	0.0146 (9)	−0.0005 (5)	0.0040 (6)	0.0009 (6)
O12	0.0191 (9)	0.0087 (7)	0.0069 (8)	−0.0009 (6)	−0.0009 (7)	0.0026 (6)

### Geometric parameters (Å, °)

**Table:**

K—O4	2.789 (2)	P1—O7	1.480 (2)
K—O5	2.860 (2)	P1—O4^vi^	1.483 (2)
K—O6^i^	2.874 (2)	P1—O12	1.588 (2)
K—O2^i^	2.961 (2)	P1—O3^iii^	1.5968 (19)
K—O10^ii^	3.075 (3)	P1—K^vi^	3.4868 (13)
K—O3^iii^	3.221 (2)	P2—O10^iv^	1.4795 (19)
K—O7^ii^	3.234 (3)	P2—O5	1.483 (2)
K—O11^iv^	3.326 (2)	P2—O11^iv^	1.6015 (18)
K—O7	3.370 (3)	P2—O3^i^	1.602 (2)
K—P3^i^	3.4008 (12)	P3—O6	1.482 (2)
K—P1^ii^	3.4868 (13)	P3—O2	1.4873 (19)
Eu—O9	2.3199 (19)	P3—O11	1.6005 (19)
Eu—O4	2.3775 (19)	P3—O1	1.6033 (19)
Eu—O10	2.3799 (18)	P3—K^vii^	3.4008 (12)
Eu—O5	2.401 (2)	P4—O9	1.4784 (19)
Eu—O7	2.4045 (19)	P4—O8^viii^	1.484 (2)
Eu—O8	2.406 (2)	P4—O1	1.601 (2)
Eu—O6^v^	2.4214 (18)	P4—O12^iv^	1.601 (2)
Eu—O2	2.4827 (19)		
			
O4—K—O5	59.57 (6)	O5—Eu—O2	141.68 (7)
O4—K—O6^i^	100.70 (7)	O7—Eu—O2	118.10 (7)
O5—K—O6^i^	89.02 (7)	O8—Eu—O2	72.99 (6)
O4—K—O2^i^	147.48 (7)	O6^v^—Eu—O2	77.51 (6)
O5—K—O2^i^	99.06 (6)	O7—P1—O4^vi^	118.08 (13)
O6^i^—K—O2^i^	51.47 (5)	O7—P1—O12	109.51 (12)
O4—K—O10^ii^	76.15 (6)	O4^vi^—P1—O12	110.78 (11)
O5—K—O10^ii^	118.26 (7)	O7—P1—O3^iii^	108.73 (11)
O6^i^—K—O10^ii^	143.04 (7)	O4^vi^—P1—O3^iii^	110.13 (12)
O2^i^—K—O10^ii^	135.75 (6)	O12—P1—O3^iii^	97.66 (11)
O4—K—O3^iii^	94.01 (6)	O10^iv^—P2—O5	121.60 (13)
O5—K—O3^iii^	93.77 (6)	O10^iv^—P2—O11^iv^	110.86 (11)
O6^i^—K—O3^iii^	164.29 (7)	O5—P2—O11^iv^	106.08 (11)
O2^i^—K—O3^iii^	112.83 (6)	O10^iv^—P2—O3^i^	107.58 (12)
O10^ii^—K—O3^iii^	46.46 (5)	O5—P2—O3^i^	109.67 (12)
O4—K—O7^ii^	49.24 (5)	O11^iv^—P2—O3^i^	98.65 (10)
O5—K—O7^ii^	108.54 (6)	O6—P3—O2	117.21 (12)
O6^i^—K—O7^ii^	97.63 (6)	O6—P3—O11	108.85 (11)
O2^i^—K—O7^ii^	138.36 (6)	O2—P3—O11	109.42 (10)
O10^ii^—K—O7^ii^	52.04 (5)	O6—P3—O1	106.70 (11)
O3^iii^—K—O7^ii^	96.13 (6)	O2—P3—O1	111.17 (11)
O4—K—O11^iv^	105.76 (6)	O11—P3—O1	102.44 (11)
O5—K—O11^iv^	46.23 (5)	O9—P4—O8^viii^	119.05 (12)
O6^i^—K—O11^iv^	78.27 (6)	O9—P4—O1	108.15 (11)
O2^i^—K—O11^iv^	57.13 (5)	O8^viii^—P4—O1	109.93 (11)
O10^ii^—K—O11^iv^	138.47 (6)	O9—P4—O12^iv^	108.86 (12)
O3^iii^—K—O11^iv^	92.53 (6)	O8^viii^—P4—O12^iv^	110.62 (12)
O7^ii^—K—O11^iv^	153.96 (6)	O1—P4—O12^iv^	98.17 (11)
O4—K—O7	55.48 (6)	P4—O1—P3	124.84 (11)
O5—K—O7	56.64 (6)	P3—O2—Eu	126.82 (12)
O6^i^—K—O7	144.25 (6)	P3—O2—K^vii^	93.80 (9)
O2^i^—K—O7	135.83 (6)	Eu—O2—K^vii^	139.28 (8)
O10^ii^—K—O7	63.35 (5)	P1^ix^—O3—P2^vii^	130.06 (14)
O3^iii^—K—O7	44.54 (5)	P1^ix^—O3—K^ix^	91.84 (9)
O7^ii^—K—O7	85.78 (3)	P2^vii^—O3—K^ix^	97.17 (9)
O11^iv^—K—O7	83.30 (6)	P1^ii^—O4—Eu	138.08 (12)
O9—Eu—O4	118.75 (7)	P1^ii^—O4—K	105.27 (10)
O9—Eu—O10	79.54 (7)	Eu—O4—K	108.28 (7)
O4—Eu—O10	142.30 (6)	P2—O5—Eu	143.49 (12)
O9—Eu—O5	71.81 (7)	P2—O5—K	110.57 (10)
O4—Eu—O5	71.94 (7)	Eu—O5—K	105.40 (8)
O10—Eu—O5	85.13 (7)	P3—O6—Eu^x^	144.03 (13)
O9—Eu—O7	138.27 (7)	P3—O6—K^vii^	97.49 (9)
O4—Eu—O7	75.03 (7)	Eu^x^—O6—K^vii^	118.48 (8)
O10—Eu—O7	70.79 (7)	P1—O7—Eu	148.46 (13)
O5—Eu—O7	76.95 (8)	P1—O7—K^vi^	87.05 (10)
O9—Eu—O8	143.51 (7)	Eu—O7—K^vi^	92.57 (7)
O4—Eu—O8	79.39 (7)	P1—O7—K	88.34 (10)
O10—Eu—O8	105.75 (7)	Eu—O7—K	91.59 (7)
O5—Eu—O8	143.72 (7)	K^vi^—O7—K	175.37 (7)
O7—Eu—O8	74.50 (7)	P4^xi^—O8—Eu	130.26 (12)
O9—Eu—O6^v^	75.16 (7)	P4—O9—Eu	146.27 (13)
O4—Eu—O6^v^	75.02 (7)	P2^iv^—O10—Eu	138.15 (12)
O10—Eu—O6^v^	142.48 (6)	P2^iv^—O10—K^vi^	106.53 (11)
O5—Eu—O6^v^	112.01 (7)	Eu—O10—K^vi^	97.15 (7)
O7—Eu—O6^v^	143.80 (7)	P3—O11—P2^iv^	132.40 (12)
O8—Eu—O6^v^	80.43 (7)	P3—O11—K^iv^	136.15 (9)
O9—Eu—O2	75.52 (7)	P2^iv^—O11—K^iv^	88.55 (8)
O4—Eu—O2	143.69 (7)	P1—O12—P4^iv^	133.66 (14)
O10—Eu—O2	69.57 (7)		

Symmetry codes: (i) *x*+1/2, −*y*+3/2, *z*−1/2; (ii) −*x*+3/2, *y*−1/2, −*z*+1/2; (iii) *x*+1, *y*, *z*; (iv) −*x*+1, −*y*+2, −*z*; (v) −*x*+1/2, *y*−1/2, −*z*+1/2; (vi) −*x*+3/2, *y*+1/2, −*z*+1/2; (vii) *x*−1/2, −*y*+3/2, *z*+1/2; (viii) *x*−1/2, −*y*+3/2, *z*−1/2; (ix) *x*−1, *y*, *z*; (x) −*x*+1/2, *y*+1/2, −*z*+1/2; (xi) *x*+1/2, −*y*+3/2, *z*+1/2.
